# Family History of Lung Cancer and Contemplation of Smoking Cessation

**Published:** 2010-02-15

**Authors:** Lisa Madlensky, Chad A. Bousman

**Affiliations:** Cancer Prevention and Control Program, Moores UCSD Cancer Center, University of California, San Diego; San Diego State/University of California, San Diego, California

## Abstract

**Introduction:**

The prevalence of cigarette smoking in the United States has decreased, but current rates remain above nationally set objectives. A family history of lung cancer may motivate adult smokers to quit and contribute to further reductions in smoking prevalence.

**Methods:**

We surveyed adult smokers (N = 838) interviewed as part of the 2005 Health Information National Trends Survey. We examined the association between family history of lung cancer and smoking cessation precontemplation (not considering), contemplation (considering), and preparation.

**Results:**

More people who reported a family history of lung cancer were in contemplation/preparation stages (41%) than were in the precontemplation stage (19%). Adults who reported a family history of lung cancer were more likely (odds ratio 2.55 [95% confidence interval, 1.44-4.52]) to be contemplators than precontemplators after adjusting for demographic variables and level of daily smoking.

**Conclusion:**

Family history of lung cancer among adult smokers may be associated with contemplating quitting smoking. Further investigation of family history's role in bolstering motivation to quit smoking may assist in developing or improving smoking cessation interventions for this group.

## Introduction

Cigarette smoking is the number 1 preventable cause of illness and death in the United States (1). Despite public health efforts, the prevalence of smoking has remained approximately 20% among US adults (2), and the *Healthy People 2010* objective to reduce the prevalence of smoking to less than 12% (3) is unlikely to be reached. Further research is required to uncover factors related to smoking behavior that can be integrated into clinical and community practice.

One potential motivator to quit smoking is family history of lung cancer. An estimated 7% to 10% of US residents have a first-degree relative with lung cancer (4,5), approximately 14% report lung cancer in any family member (6), and among nonsmokers, 9% report a family member with lung cancer (7). Family history can help people make health-related decisions. Previous research shows that family history of several chronic diseases (eg, heart disease, diabetes) may increase motivation for health screening behaviors (8-10), daily consumption of fruits and vegetables (8), and perceived susceptibility to health consequences (11). It is not known whether family history of lung cancer is a viable correlate of contemplating or preparing to quit smoking.

The stages of change identified in the transtheoretical model (12) are 1) precontemplation, 2) contemplation, 3) preparation, 4) action, and 5) maintenance. People progress through the cycle of change at different rates, from not considering change (precontemplation) to considering change (contemplation), planning for change (preparation), making changes (action), and sustaining changes (maintenance).

According to the transtheoretical model, treatment providers can help smokers through these stages by influencing decisional balance (ie, pros and cons) and self-efficacy for quitting and by applying or engaging them in catalysts for change (13,14). For smokers, consciousness-raising increases awareness of the causes and consequences of smoking (12,15,16). Consciousness-raising is most appropriate in the early stages of change and is less advantageous in later stages (17). In this retrospective study, we hypothesized that people with a self-reported family history of lung cancer would be more likely to belong in the contemplation and preparation stages than would people without a family history of lung cancer among a nationally representative sample of adult smokers.

## Methods

### Data source

For this study, we used cross-sectional data collected as part of the National Cancer Institute 2005 Health Information National Trends Survey (HINTS) (6). From February 2005 through August 2005, surveyors used a random-digit–dialing approach to select 5,586 US civilian, noninstitutionalized adults for a single telephone or Web-based interview following the best practices identified by the American Association for Public Opinion Research (18). The Web-based option was first offered in 2005 as an attempt to improve low response rates observed in the 2003 HINTS. Response rates for the 2005 screener questionnaire and full interview were 34% and 61%, respectively (for additional information, visit http://cancercontrol.cancer.gov/hints/). For this study, we selected participants if they met the following criteria: 1) reported ever smoking 100 cigarettes or more in their lifetime, 2) identified as a current smoker, 3) did not have a personal history of any cancer, and 4) were not missing data on smoking or family history variables.

Of the eligible 5,586 adults surveyed, surveyors asked 5,505 if they smoked 100 or more cigarettes in their lifetime, and 2,615 responded affirmatively. Of these participants, 1,015 (18% of 5,505 surveyed) identified as a current smoker and 864 reported no personal history of any cancer. An additional 26 people were excluded because of missing data for smoking or family history variables required for hypothesis testing, resulting in a total sample of 838 participants. Participants excluded as a result of missing data were not demographically different from the selected study sample. The University of California, San Diego, institutional review board reviewed and approved this study.

### Smoking-related variables

We classified participants as current smokers if they responded "every day" or "some days"  to a question on smoking frequency: "Do you now smoke cigarettes every day, some days, or not at all?"  Current smokers were further asked: "Are you seriously considering quitting smoking within the next 6 months?" Smokers answering no were classified as "precontemplators" (n = 247). Among smokers answering yes to considering quitting, we examined their responses to the following question: "How many times during the past 12 months have you stopped smoking for 1 day or longer because you were trying to quit smoking?" We classified participants reporting 1 or more quits (stopped smoking for 1 day or longer because they were trying to quit smoking) as being in the preparation stage (n = 355), whereas we considered those reporting zero quits to be in the contemplation stage (n = 236). Because of the nature of the sample (current smokers), we did not use the other stages.

To estimate degree of nicotine dependence, we created a variable summarizing the number of cigarettes smoked per day. For "every day" smokers we used the following question: "On average, how many cigarettes do you smoke a day?" For "some day" smokers we used 2 questions: "On how many of the past 30 days did you smoke cigarettes?" and "On average, on those days, how many cigarettes did you usually smoke each day?" To estimate cigarettes per day for "some day" smokers, we multiplied the number of days smoking by the average number of cigarettes on those days; we divided the product by 30.

### Family history of lung cancer

All participants were asked: "Have any of your family members ever had cancer?" If they answered yes, we then asked them what type of cancer each diagnosed family member had; however, we did not collect data on the relationship between the diagnosed family member and participant. Participants reporting a family member diagnosed with lung cancer were classified as family history positive and those reporting no lung cancer among family members were classified as family history negative.

### Other variables analyzed

Demographic characteristics measured included sex, age, education, and race/ethnicity. We measured age continuously in years and recoded education into a binary variable, "12 years or less" and "13 years or more." We also collapsed race/ethnicity into a binary variable in which non-Hispanic whites (69%) were compared with nonwhites (31%). Nonwhites included Hispanic (11%), African American (11%), American Indian (4%), Asian (1%), and other (4%).

### Sample weighting and statistical analysis

Person-level survey weights were developed for the 2005 HINTS, so that estimates are representative of the US adult population (for detailed information, visit http://cancercontrol.cancer.gov/hints/). All statistical tests and procedures used weighted estimates computed in STATA's 2005 svy package version 10 (StataCorp LP, College Station, Texas). We used the Pearson χ^2^ statistic to explore the unadjusted associations between family history of any cancer as well as lung cancer and stage of change for quitting smoking. We used logistic regression models to compare stages of change (contemplation vs precontemplation and preparation vs contemplation). We selected each potential covariate (ie, family history of lung cancer, age, sex, ethnicity, education, smoking frequency) for its documented association with the intention to quit smoking (15) as well as smoking cessation (19) in the literature. Differences were considered significant at *P < *.05.

## Results

### Family history and stage of change

We examined the association between family history of lung cancer and quitting stage of change (Figure 1). Nearly one-quarter (24% [n = 202]) of the adults sampled in this study reported having at least 1 family member diagnosed with lung cancer. Half (51% [n = 429]) of the participants reported a family history of cancer other than lung cancer and the remaining one-quarter (25% [n = 207]) reported no family history of any cancer. Most of those with a family history of lung cancer were in the preparation stage (44%) for quitting smoking, followed by the contemplation (37%) and precontemplation (19%) stages. These proportions differed significantly (*χ* ^2^ = 19.62, *df* = 4, *P* = .02) from those for adults with a family history of cancer other than lung cancer and those who reported no history of any cancer. Specifically, we observed significant proportional differences in the precontemplative (*χ* ^2^ = 18.21, *df* = 2, *P* < .001) and contemplative (*χ* ^2^ = 10.12, *df* = 2, *P* = .006) stages but not the preparation stage (*χ* ^2^ = 2.52, *df* = 2, *P* = .28). Furthermore, in each of the 3 stages, the proportion of participants with a family history other than lung cancer and those without a family history did not differ significantly (*χ* ^2^ = 1.50, *df* = 2, *P* = .47).

**Figure.**
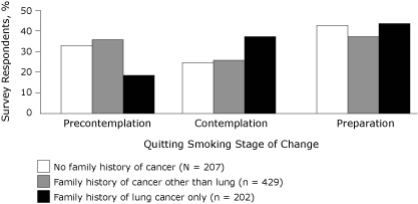
Most of the adults sampled in this study who had a family history of lung cancer were in the preparation stage (44%) for quitting smoking, followed by the contemplation (37%) and precontemplation (19%) stages.

**Table:** 

	**No family history of cancer (N = 207)**	**Family history of cancer other than lung (n = 429)**	**Family history of lung cancer only (n = 202)**
Precontemplation	33%	36%	19%
Contemplation	25%	26%	37%
Preparation	43%	38%	44%

### Predictors of stage of change

We used 2 multivariate logistic regressions that modeled contemplation vs precontemplation and preparation vs contemplation (Table). Both models included the family history of lung cancer and the aforementioned covariates based on past research (15,19). In the precontemplation vs contemplation stage model, we found that adults reporting a family history of lung cancer were 2.55 (95% confidence interval [CI] 1.44–4.52) times as likely to be classified as contemplators after adjusting for other covariates. However, family history of lung cancer did not differ for adults in the preparation vs contemplation stage model (adjusted OR, 0.75; 95% CI, 0.45–1.24).

## Discussion

Having a family member with lung cancer was not associated with preparing to quit smoking. Adults who reported a family history of lung cancer were 2.55 times as likely to be in the contemplation stage than in the precontemplation stage; we found no difference between the contemplation and preparation stages. Thus, our results do not suggest that having a family history of lung cancer necessarily leads to more quit attempts or increases the success of cessation. In fact, the transtheoretical model would posit that consciousness-raising resulting from family history is not sufficient for behavioral change and must be employed along with 9 other catalysts of change to help smokers quit. For this reason, translation of these findings into practice is premature and requires longitudinal research to determine the underlying mechanisms (eg, perceived risk) by which this observed association is operating. However, given that family history predicts disease-specific perceived risk (20) and is associated with other health behaviors, particularly those with a physician gatekeeper (eg, colorectal cancer screening [21], breast cancer screening [22], and aspirin use or cholesterol screening [9]), the observed association may provide insight into the complex behavior of smoking cessation.

Although these findings are intriguing, they should be viewed as preliminary and be weighed against several limitations. We used a representative sample, which allows results to be generalized to the population level; however, response rates were lower than ideal (61%) and as a result may bias against those of low socioeconomic status or from minority population groups. Although the data are weighted to adjust for this underrepresentation, this does not completely remove the potential bias. Furthermore, the measure of family history of cancer was nonspecific, and we were unable to ascertain the relationship of the family member to the respondent (eg, first-degree vs second-degree relative), the number of family members diagnosed with lung cancer, or when the family member was diagnosed with lung cancer. Having access to these types of information would allow for more specificity in estimating the association between family history of lung cancer and smoking cessation contemplation and preparation. However, regarding who in the family was diagnosed with lung cancer, the risks of continued smoking may be more salient for participants with first-degree relatives (mother, father, and sibling) than for those whose more distant relatives had lung cancer. Thus, our results may represent a conservative estimate of the association between family history of lung cancer and contemplating and preparing to quit smoking.

In addition, we were unable to assess whether the relative with lung cancer was also a smoker or whether other family members smoked but never had lung cancer. Thus, we lack a measure of the salience of the link between smoking and risk of lung cancer to each participant. Also, we note that most smokers do not report a family history of lung cancer (76% in this study) and thus the observed association may not be applicable for most smokers. Finally, because data for this study were cross-sectional, we cannot determine the temporal order of the relationships described. Smokers in the contemplation stage may have been more aware of their family history as a result of their contemplation to quit; if so, we may be reporting an effect-cause relationship. Future longitudinal studies among smokers who have never reported contemplating quitting smoking could further clarify the direction of this relationship.

Family history of lung cancer among adult smokers appears to have a significant association with contemplation of smoking cessation. The continued stability of smoking rates among US adults indicates that novel approaches in clinical and community settings are needed to decrease smoking prevalence. Future investigation examining the role of family history in bolstering motivation to quit smoking may be helpful.

## Figures and Tables

**Table.** Association Between Awareness of Family History of Lung Cancer and Stage of Change for Quitting Smoking Among Current Smokers (N = 838)

**Variable**	C vs PC (n = 483), OR (95% CI)	P vs C (n = 591), OR (95% CI)
**Family history of lung cancer**		
Positive (n = 202)	2.55 (1.44-4.52)	0.75 (0.45-1.24)
Negative (n = 636)	1 [reference]	1 [reference]
**Age, y (n [SD] = 44 [15])**		0.99 (0.98-1.01)
**Ethnicity**		
Nonwhite (n = 228)	0.76 (0.43-1.36)	1.31 (0.80-2.13)
White (n = 610)	1 [reference]	1 [reference]
**Sex**		
Female (n = 494)	1.14 (0.71-1.82)	0.94 (0.62-1.45)
Male (n = 344)	1 [reference]	1 [reference]
**Education**		
>13 y (n = 404)	0.88 (0.53-1.43)	1.25 (0.80-1.97)
0-12 y (n = 434)	1 [reference]	1 [reference]
**Cigarettes per day **(n = 14)	0.97 (0.95-0.99)	1.02 (1.01-1.05)

Abbreviations: PC, precontemplation; C, contemplation; P, preparation.
